# Using a programmatic mapping approach to plan for HIV prevention and harm reduction interventions for people who inject drugs in three South African cities

**DOI:** 10.1186/s12954-017-0164-z

**Published:** 2017-06-07

**Authors:** Andrew Scheibe, Shaun Shelly, Andrew Lambert, Andrea Schneider, Rudolf Basson, Nelson Medeiros, Kalvanya Padayachee, Helen Savva, Harry Hausler

**Affiliations:** 1 TB/HIV Care Association, Cape Town, South Africa; 2 Desmond Tutu HIV Centre, Cape Town, South Africa; 3 OUT LGBT Wellbeing, Pretoria, South Africa; 4 United States Centers for Disease Control and Prevention, Pretoria, South Africa; 5 School of Public Health, University of the Western Cape, Cape Town, South Africa; 6 Department of Psychiatry and Mental Health, University of Cape Town, Cape Town, South Africa

**Keywords:** Formative assessment, People who inject drugs, Harm reduction, HIV, South Africa, Population size estimation, Programmatic mapping

## Abstract

**Background:**

Stigma, criminalisation and a lack of data on drug use contribute to the “invisibility” of people who inject drugs (PWID) and make HIV prevention and treatment service delivery challenging. We aimed to confirm locations where PWID congregate in Cape Town, eThekwini and Tshwane (South Africa) and to estimate PWID population sizes within selected electoral wards in these areas to inform South Africa’s first multi-site HIV prevention project for PWID.

**Methods:**

Field workers (including PWID peers) interviewed community informants to identify suspected injecting locations in selected electoral wards in each city and then visited these locations and interviewed PWID. Interviews were used to gather information about the accessibility of sterile injecting equipment, location coordinates and movement patterns. We used the Delphi method to obtain final population size estimates for the mapped wards based on estimates from wisdom of the crowd methods, the literature and programmatic data.

**Results:**

Between January and April 2015, we mapped 45 wards. Tshwane teams interviewed 39 PWID in 12 wards, resulting in an estimated number of accessible PWID ranging from 568 to 1431. In eThekwini, teams interviewed 40 PWID in 15 wards with an estimated number of accessible PWID ranging from 184 to 350. The Cape Town team interviewed 61 PWID in 18 wards with an estimated number of accessible PWID ranging between 398 and 503. Sterile needles were only available at one location. Almost all needles were bought from pharmacies. Between 80 and 86% of PWID frequented more than one location per day. PWID who reported movement visited a median of three locations a day.

**Conclusions:**

Programmatic mapping led by PWID peers can be used effectively to identify and reach PWID and build relationships where access to HIV prevention commodities for PWID is limited. PWID reported limited access to sterile injecting equipment, highlighting an important HIV prevention need. Programmatic mapping data show that outreach programmes should be flexible and account for the mobile nature of PWID populations. The PWID population size estimates can be used to develop service delivery targets and as baseline measures.

## Background

“After 30 years the time for dealing with injecting drug use has finally come,” proclaimed Dr. Fareed Abdullah, the then Chief Executive Officer, South African National AIDS Council. This address at the South African Drug Use and HIV Preconference in 2015 highlighted the dearth of HIV prevention services for people who inject drugs (PWID) in South Africa [1].

### Injecting drug use and HIV

PWID are at disproportionately higher risk for HIV infection and onward transmission compared to the general population [2]. HIV is efficiently spread through the use of contaminated injecting equipment and can spread rapidly within PWID networks [3, 4]. PWID are also at risk of HIV transmission through unprotected sex, which is influenced by links to sex work and drug-related effects on decision making [5, 6].

Several social and structural factors contribute to the increased HIV burden among PWID. For example, the criminalisation of drug use contributes to the marginalisation and social exclusion of PWID, which increases their vulnerability and often creates barriers to effective provision of and access to health services, particularly in the public sector [7]. Furthermore, injecting drug use often occurs covertly or in dangerous environments (such as in dilapidated buildings or tunnels) and rapidly (for fear of being arrested by the police)—indirectly increasing the health-related risks of injecting through using contaminated needles and incurring wounds through rapid injecting practices [8]. Stigma and discrimination by community members and health providers contribute to delayed health-seeking behaviours among PWID [9]. Furthermore, a lack of appropriate HIV prevention, treatment, care and support services contribute to the HIV burden and risk of onward HIV transmission among PWID, their sexual and drug-using partners and the broader population [10]. Limited data on injecting drug practices, particularly in Africa, contribute to the populations’ “invisibility” [11]. The social and structural factors that increase the risk of HIV among PWID are important in the African context where the HIV prevalence is high, and access to evidence-based interventions is limited.

### Injecting drug use and HIV in South Africa

Drug use is criminalised in South Africa, but no laws prohibit the purchase or provision of injecting equipment. South Africa’s national PWID population size is measured by a modelling study using information from the 2008 South African household survey data; this study estimates that there are 67,000 PWID in South Africa [12]. Sub-national or city-specific estimates do not exist, limiting the ability to appropriately plan and provide services for PWID. However, local research has identified the use of contaminated injecting equipment, unprotected sex, sex work and low levels of HIV and drug-related knowledge among PWID in South Africa [13–17]. In 2013, HIV prevalence among PWID participating in a multi-city study, including Cape Town, eThekwini and Tshwane (*n* = 450) was 14% [17]. The existing data point to high-risk practices and the potential of ongoing transmission unless effective prevention interventions become available.

### HIV prevention and treatment services for PWID in South Africa

Substance-use disorder treatment and HIV services for PWID in South Africa are limited and are primarily provided by civil society organisations. Publicly funded substance-use disorder treatment services are almost exclusively abstinence-based (i.e., organisations that view abstinence of drug use as the only treatment outcome with no interventions to reduce the potential harms of drug use). Private sector services are prohibitively expensive for most PWID [17–20]. None of the existing service providers provide the full package of evidence-based HIV prevention, treatment and care services for PWID as recommended by the World Health Organization (WHO)/United Nations Office on Drugs and Crime (UNODC)/Joint United Nations Programme on HIV and AIDS (UNAIDS) and President’s Emergency Plan for AIDS Relief (PEPFAR). In 2016, opioid substitution therapy (OST), a key WHO/UNODC/UNAIDS/PEPFAR-recommended intervention, was only available free of charge through one civil society organisation based in Cape Town. The project provides OST for free for 6 months and has provided OST to 178 people (almost exclusively non-injecting heroin users) between 2013 and 2016 [21]. Although needle and syringe programmes are evidence-based and are also part of the earlier mentioned WHO/UNODC/UNAIDS/PEPFAR-recommended package, only one programme targeting men who have sex with men who inject drugs operated in Cape Town for a limited period (September 2013 to November 2014) [22, 23]. In a country with the highest HIV burden and a substantial PWID population, it is clear that two programmes for PWID are insufficient.

The overall goal of the programmatic mapping activity was to prepare three sites to establish PWID HIV prevention and harm reduction services. The study objectives were to (1) map locations where PWID congregate in selected electoral wards in three cities, (2) collect information to inform the delivery of HIV prevention and harm reduction services for PWID and (3) estimate the number of PWID accessible at identified locations in selected electoral wards in the selected cities.

## Methods

**Fig. 1:**
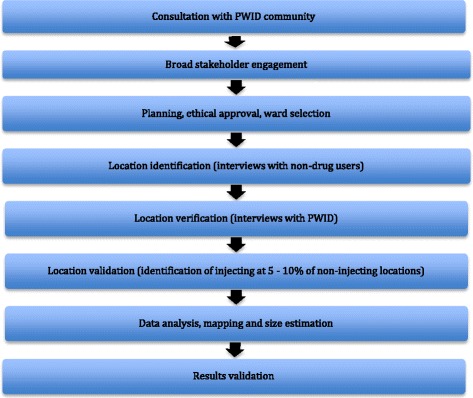
Overview of formative assessment methodology

This study employed a modified programmatic mapping approach [24]. An overview of the study methods are summarised in Fig. 1.

### Formative assessment team

Teams included a coordinator, field workers (including PWID peers), data capturers, a driver and support staff. In addition, PWID volunteers worked as guides to facilitate links with other PWID community members. Team members and PWID volunteers were selected based on their understanding of the local drug-using context and interest in being involved in the project. A central management team oversaw the planning and implementation.

### Stakeholder engagement

During the first half of 2014, the authors and representatives from their organisations held consultation workshops with PWID in Cape Town, eThekwini and Tshwane. Approximately 20 PWID attended each workshop. PWID community advisory groups (CAGs) comprised of interested PWID were established. Participants were reimbursed for their transport to the workshop and CAGs (ZAR 40 (US$2.91) and ZAR60 (US$4.36),^1^ respectively). In early 2014, we conducted stakeholder workshops with representatives from law enforcement agencies, health and substance-use disorder treatment providers, government representatives and other stakeholders. These workshops led to stronger relationships with the PWID community and other stakeholders, which allowed successful study implementation and later service delivery.

### Ward selection

Study staff mapped locations where PWID were thought to congregate onto large city maps. These maps included electoral ward boundaries that are outlined by South Africa’s municipal demarcation board [25]. Each site team developed a list of wards to be mapped. Wards were ranked by the number of suspected PWID locations in each ward, which was informed by the earlier consultations. The number of wards (18 in Cape Town, 16 in eThekwini and 12 in Tshwane) was determined by team size, budget and timeframe.

### Training

Study teams were trained on the methodology of the formative assessment, personal safety, ethical research practice and human subjects’ protection, engagement strategies, data entry and quality control over a period of 3 days. Training included didactic lectures, interactive sessions and role-plays.

### Fieldwork

In December 2015, we piloted tools and fieldwork activities in wards not selected for formal mapping. Formative assessment teams met at the beginning of the day to plan activities and at the end of the day to discuss outcomes and challenges. Site teams engaged with the central management team each week. Teams reflected on the various stages of the formative assessment, including successes and challenges; these reflections were used to inform planning for service delivery and engagement with the PWID community and stakeholders. Fieldwork included location identification (LI), location verification (LV) and location validation activities.

#### Location identification

Working in pairs, fieldworkers conducted up to 20 brief interviews per day with secondary key informants (community members who were not drug users) in selected wards. Secondary key informants were chosen because they had some level of engagement or knowledge of drug use in their community. The type of secondary key informants approached were identified as part of earlier consultation processes and included street vendors, taxi drivers, shop keepers and law enforcement officers. Interviews lasted approximately 10 min each. After introducing themselves, team members described the rationale, objectives and nature of the brief interview and the study, provided interviewees with an information sheet and answered questions before asking for verbal informed consent. Interviews were undertaken in local languages (i.e. English, Afrikaans, isiZulu, Setswana and isiXhosa). Fieldworkers started by asking secondary key informants “what the situation with drug use was like in the area” and “where can people who use drugs be found?” Locations included places where PWID (i) bought, sold and/or used drugs; (iii) engaged in income generating activities; (iii) socialised and (iv) slept. Fieldworkers then probed for names and addresses of these locations. Additional questions were asked about each location (e.g. the number of people who use drugs at these locations and their sex and whether injecting drug use occurred at these locations). Participants were offered condoms and lubricants once the interview was completed. HIV risk reduction counselling was provided where possible and appropriate. Fieldworkers reconvened at the end of each day to collate data and to generate lists of locations.

#### Location verification

Fieldworkers visited all locations that were identified by at least one secondary key informant as being a location where PWID possibly congregate. Fieldworkers recorded location coordinates of all locations visited using a global positioning system (GPS) device. At each location, fieldworkers attempted to identify and engage with an injecting drug user (primary key informant). Additional information about the location and visibility of HIV prevention commodities (unused condoms, sealed lubricant and unused injecting equipment) was recorded. Verification interviews lasted approximately 10 min each and followed a similar format to the location identification process (i.e. introduction, project overview, verbal consent and specific questions). Outreach teams revisited sites up to three times if a primary key informant was not identified, but injecting equipment was visible or a non-injecting drug user confirmed that PWID had been seen at that location. PWID interviews covered details of (1) the location (busiest time, number and types of PWID that congregate there, location of nearest clinic, access to condoms and lubricants, needle and syringe access and disposal, drug sales, sex work and experiences with law enforcement); (2) mobility (average number of locations PWID meet or congregate at during one day); (3) perceptions about whether their health needs were being met; and (4) details of other locations where PWID congregate.

#### Location validation

The research team visited a subset^2^ of locations that were identified by secondary key informants as locations where people use (but not inject) drugs to assess underestimation of PWID locations. Validation visits followed the location verification procedures, and efforts were made to identify and interview PWID.

#### Results validation

Fieldwork findings were presented to the PWID CAGs in September 2015. CAG members’ input on the findings was obtained, specifically around the identified locations, average numbers of estimated PWID at identified locations and PWID movement.

### Data analysis, mapping and size estimation

#### Data analysis

Data from the location identification and verification forms were entered into a password-protected excel spreadsheet. Data was then imported into Stata (version 11.0, STAT Corp., College Station, Texas, USA) for analysis. We calculated measures of central tendency and dispersion for numerical variables and used frequency tables to explore categorical data.

#### Geospatial mapping

We exported GPS coordinates of suggested PWID locations into an excel spreadsheet. These GPS coordinates and details about location type (i.e. whether or not PWID were interviewed) were entered into QGIS (version 2.6.1 Brighton). Finally, we mapped the locations that were visited graphically to inform outreach service delivery routes. To ensure confidentiality and to safeguard PWID communities included in the mapping, no location details are presented in this manuscript.

#### Size estimation

We developed six PWID estimates using different methods:

1. ‘Location verification estimates’ reflected data collected during location verification and location validation processes as per a formula adapted from previous programmatic mapping studies, which take PWID movement into account [24] (see Fig. 2).

**Fig. 2:**
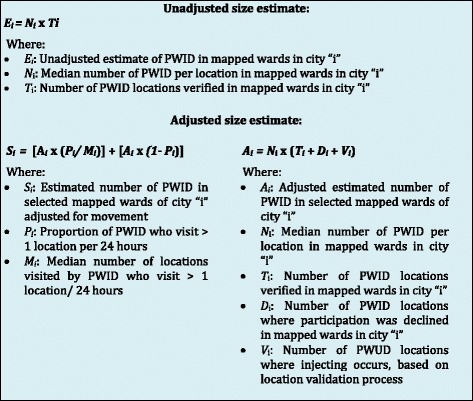
Formula for developing size estimates based on location verification data
2. ‘CAG revised location verification finding estimates’ were revisions of the initial estimates based on input provided by the CAGs at results validation meetings.
3. ‘CAG estimates’ are the estimates of the number of PWID who CAGs estimated to be accessible in the mapped wards in each city using consensus estimation methods [26]. These were also conducted during the result validation meetings.
4. ‘Literature estimates’ were developed for each mapped ward. The number of people aged 18 to 64 years for each ward (based on 2011 census data) [27] was multiplied by 0.2%, the estimated prevalence of injecting drug use from an earlier South African study [12].
5. ‘Service delivery consent form estimates’ were obtained by counting the number of PWID who had accessed needle and syringe and other HIV prevention^3^ services between June and October 2015 and that consented for service delivery data to be used.
6. ‘Service delivery contact form estimates’ were obtained by counting the number of PWID who had accessed needle and syringe and other HIV prevention services. Individuals were tracked with unique participant identification codes (recorded on outreach contact forms) between June and October 2015.

The final population size estimate reflects an estimated range of PWID that could be reached with HIV prevention services in the mapped wards.

#### Quality assurance

Assessment procedures were standardised, and scripts were developed and used for relevant study procedures. Trained staff, fluent in local languages, completed study activities. Research team members observed interviews and used a standardised checklist to monitor interviews.

### Ethical considerations

The University of the Western Cape (reference number 14/3/9), the KwaZulu-Natal Provincial Department of Health (HRKM 01/15) and the United States Centers for Disease Control and Prevention (PR 2013-38) provided ethical approval for this study.

## Results

The results of the formative assessment are provided by city, including details of PWID locations, service delivery needs and size estimates. An overview of formative assessment participants and characteristics of the confirmed locations where PWID congregated in each city is shown in Table 1.

**Table 1:** Demographic characteristics of PWID interviewed and characteristics of locations where PWID congregate in selected wards in Cape Town, Tshwane and eThekwini, 2015

Metropolitan area		Cape Town	Tshwane	eThekwini
		*n*	*n*	*n*
No. of mapped electoral wards		18	12	15^a^
No. of secondary key informants interviewed		400	280	385
No. locations where drug use suspected		734	146	222
No. locations where injecting suspected		230	68	164
No. locations where PWID interviewed		61	36	40
Locations where injecting is not suspected but PWID identified^b^		0/29 (0%)^c^	1/13 (8%)	1/14 (7%)
Median no. of people estimated to congregate at locations (interquartile range)		7 (4–15)	10 (5–13)	6 (3–11)
		*n* (%)	*n* (%)	*n* (%)
Sex of PWID interviewed	Male	56 (92%)	31 (86%)	35 (88%)
	Female	5 (8%)	5 (14%)	5 (12%)
Race of PWID interviewed	Black	3 (5%)	16 (44%)	6 (15%)
	White	22 (36%)	18 (50%)	29 (73%)
	Coloured^d^	36 (59%)	2 (6%)	1 (3%)
	Indian/Asian	0	0	4 (10%)
No. of locations where commodities seen	Condoms	3 (5%)	0	1 (3%)
	Lubricant	2 (3%)	0	0
	Sterile injecting equipment	0	0	1 (3%)
No. of locations where sterile injecting equipment reportedly available (for purchase)		3 (5%)	6 (17%)	6 (15%)
Needle and syringe disposal	Discarded in bin, needle intact	26 (43%)	16 (44%)	20 (51%)
	Needle broken off, then discarded in bin	11 (18%)	11 (31%)	10 (26%)
	Needle discarded on the ground	11 (18%)	5 (14%)	7 (18%)
Sex work occurs at the location		33 (54%)	15 (42%)	23 (58%)
Drug sales/purchase occurs at the location		37 (61%)	26 (72%)	23 (58%)

^a^Mapping of one ward was not possible due to social unrest

^b^These locations and PWID are included in the total number of PWID interviewed and verified PWID locations

^c^Four locations were not visited due to violence in the area

^d^‘Coloured’ is a recognised South African racial group and refers to people of mixed Black/African, European and/or Asian ancestry

### Cape Town

#### PWID locations

We interviewed 399 secondary key informants (262 males, 131 females, and 6 transgender women) in 18 of Cape Town’s 111 electoral wards. Of the 230 suggested PWID locations, the team visited and identified 69 locations where at least 1 PWID was present. PWID at eight of these locations refused participation in the study. Sixty-one PWID were interviewed at 61 locations (56 males and 5 females). Safety concerns linked to gang violence prevented the team from visiting 37 potential PWID locations and 4 locations selected for location validation. Most PWID congregated in public spaces, near bridges, parks, or on the street (61%, 37/61). No PWID were identified at the 29 non-injecting locations that were selected and visited as part of the location validation process.

#### Service delivery needs and other location characteristics

Sterile injecting equipment was not reportedly available at any of the PWID locations. Sex work and drug sales took place at more than half of the PWID locations (54% (33/61) and 61% (37/61), respectively). Two-thirds of PWID (56/91) could identify nearby health facilities, and 58 identified locations where condoms could be accessed. Almost all PWID (92%, 56/61) identified pharmacies where needles and syringes could be purchased. Less than half of PWID (38%, 23/61) felt that their health needs were met. Breaking used needles and then discarding them in the rubbish bin was the most common method of needle and syringe disposal (43%, 26/61). Most (84%, 51/61) PWID visited four locations per day where other PWID congregated. Law enforcement was the major cause of PWID movement.

#### Population size estimate

**Fig. 3:**
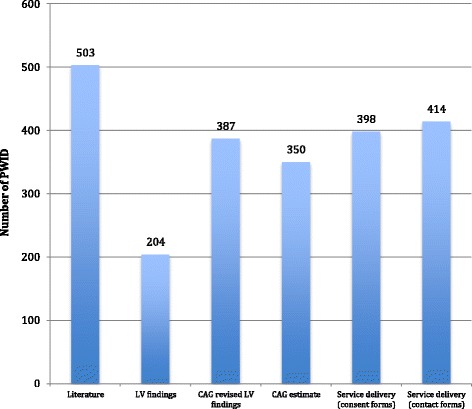
PWID estimates by data source, 16 electoral wards mapped in Cape Town, 2015

We found that on average, seven PWID congregated at each location at peak times. The range of PWID estimated to be accessible in the 18 mapped wards, as per final consensus, was 398 to 503 (see Fig. 3).

### Tshwane

#### PWID locations

We interviewed 280 non-drug users (221 males and 59 females) in 12 of Tshwane’s 105 electoral wards. Of the 90 suggested PWID locations, 37 PWID locations were verified and one consenting PWID interviewed at each of 35 locations. The team identified and interviewed an additional PWID at one of the 13 non-injecting locations selected to be visited as part of the location validation process. In total, 31 male and 5 female PWID were interviewed. Most locations (92%, 33/36) were in public spaces, including on the street, near bridges and around parks.

#### Service delivery needs and other location characteristics

Sterile injecting equipment was not reportedly available at any of the PWID locations, but was reportedly available nearby (i.e. could be purchased from a pharmacy or drug dealer) at six locations. Sex work occurred at just less than half (42%, 15/36) of the locations, and drug sales occurred at about three quarters (72%, 26/36) of the locations. Most PWID (83%, 30/36) identified local health facilities. Almost all PWID (92%, 33/36) identified local pharmacies where needles and syringes could be purchased. Two-thirds (69%, 25/36) of PWID identified locations where condoms were accessible. A quarter of PWID (25%, 9/36) felt that their health needs were being met. Discarding used needles and syringes in the rubbish bin was the most common method of needle and syringe disposal (44%, 16/36). Most PWID (86%, 31/36) visited approximately three locations per day. Law enforcement was the major cause of PWID movement.

#### Population size estimate

**Fig. 4:**
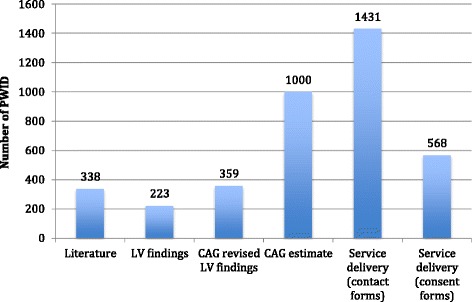
PWID estimates by data source, 12 electoral wards mapped in Tshwane, 2015

Ten PWID usually congregated at each of the identified locations at the busiest time. The range of PWID estimated to be accessible in the 12 mapped wards was 568 to 1431 (see Fig. 4).

### eThekwini

#### PWID locations

We interviewed 384 non-drug users (283 males and 101 females) in 15 of eThekwini’s 110 electoral wards. One ward could not be mapped due to social unrest, which included violent protests. Ninety PWID locations were suggested in total. A total of 39 suggested PWID locations were visited. All PWID who were approached agreed to participate. Injecting was identified at one of the 14 non-injecting locations that were visited as part of the location validation process. Forty interviews (35 male and 5 female PWID) were conducted in total. Most locations (70%, 28/40) were in public spaces including on the street, near bridges, parks and public toilets.

#### Service delivery needs and other location characteristics

Sterile injecting equipment was reportedly available for purchase at one location and near to six other locations. Sex work and drug sales occurred at more than half of the locations (58% (23/40) and 58% (23/40), respectively). All PWID identified local health facilities, and 34 identified locations where condoms could be accessed. Almost all PWID (98%, 39/40) identified local pharmacies where needles and syringes could be purchased. Five PWID felt that their health needs were being met. Discarding used injecting equipment into the rubbish bin was the most common method of disposal (50%, 20/40). Most (83%, 33/40) PWID visited approximately three PWID locations per day. Most PWID moved for drug-related reasons (i.e. to purchase drugs or to conduct activities (e.g., begging, doing casual jobs) to obtain money to be able to acquire drugs).

#### Population size estimate

**Fig. 5:**
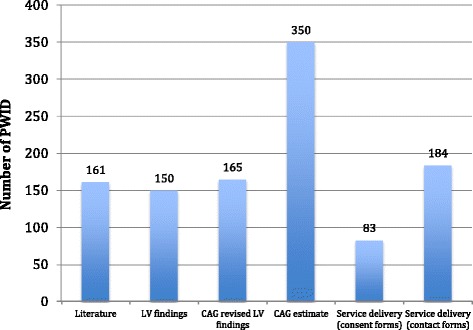
PWID estimates by data source, 15 electoral wards mapped in eThekwini, 2015

Six PWID usually congregated at each of the PWID locations at the busiest time. The range of PWID estimated to be accessible in the 15 mapped was 184–350 (see Fig. 5).

## Discussion

This is the first study that maps locations where PWID congregate in three of South Africa’s major cities. In general, secondary key informants were not able to identify locations where injecting took place, either overestimating the number of locations where injecting occurred or not being aware of other locations where PWID congregate. Our research confirmed injecting drug use at less than half of the suggested locations. Furthermore, injecting drug use was confirmed at 4% of locations where secondary key informants did not suspect injecting drug use.

Across all cities, PWID were accessible and congregated in public spaces. This is to be expected and is described in the literature and ascribed to easy access to both consumers and dealers [28], safety-seeking [28, 29], income generation [30] and socialisation [29]. The complex nexus of police action and ability to practice harm reduction in public spaces has also been described in the literature [31, 32]. Sterile injecting equipment was only available at one of these locations, highlighting the lack of outreach services, which could provide education, agency development and harm reduction equipment. Previous research among PWID in these cities found that half of PWID reused their injecting equipment the last time they injected and more than half had ever shared injecting equipment with someone else [17]. The limited access to injecting equipment is likely to be a contributing factor for needle and syringe reuse and sharing in these cities.

Although most PWID knew where their local clinic was located, or where needles and syringes could be purchased, only a third felt that their health needs were being met. This finding is unsurprising as HIV prevention and outreach have not focused on PWID in either Tshwane or eThekwini during the time the study took place, and a short-term harm reduction programme for men who have sex with men that included a needle and syringe component was provided in Cape Town [23].

Sex work and drug sales commonly occurred at about two-thirds of the locations where PWID congregate. Previous studies among PWID in South Africa have identified sex work among PWID, particularly among female PWID [17, 33, 34]. The intersection between drug use and sex work is important from an HIV point of view, which service providers should consider when planning services. This study did not assess PWID’s engagement in sex work.

Few female PWID were recruited as part of the assessment. This recruitment pattern reflects previous South African studies that have also suggested that women who inject drugs face additional stigma and discrimination and that their specific health and social needs have not been met [17, 35].

The PWID in these cities are mobile, with movements often influenced by their engagement with law enforcement officers. This finding confirms similar reports noting the need to improve relationships between PWID and law enforcement officers, with a move away from criminalising people who use drugs [36–39] and exploring options of police assisted referral to harm reduction services [40]. International data from various settings highlights that fear of and experienced negative engagement with police contributes to rushed and unsafe injecting practices [30, 41–44]. The role of private security, who play an integral part of South Africa’s security sector, has also been described as having a negative impact on access to health services and the rights of PWID [45]. Local data describing and quantifying the contribution of law enforcement on injecting-related risk among PWID in South Africa is limited. However, this study notes that engagement between PWID, law enforcement, mobility, and related risk are core considerations when designing services for PWID. International experience has shown that an enabling environment for improved public health is possible through partnerships with police [46–48].

In addition to limited access to injecting equipment and frequent encounters with police, PWID at these locations reported unsafe needle and syringe disposal practices. Many PWID disposed of used injecting equipment in ways that they thought were safe (e.g. breaking off needles). However, many PWID reported disposing needles in garbage collection facilities or in public spaces. This finding is not surprising given the absence of needle and syringe programmes or other systems for the safe disposal of sharps in these cities. Creating mechanisms for safe disposal of injecting equipment should also be a core element of needle and syringe programmes, which would be more effective with police support [49].

The methods we used in this study provided insights into locations where PWID congregate and what services are needed and highlighted important considerations when planning services for PWID. Security threats and the clandestine nature of PWID networks were challenges that we needed to overcome to complete the study, and these same challenges could be considered when planning HIV prevention and related services for PWID.

Reflection on the study teams’ experience of the formative assessment highlighted critical issues that should be considered when designing PWID services. Several team members reported the assessment as being an emotionally challenging experience; many felt negatively affected by their exposure to the high burden of social and health issues experienced by PWID in the mapped areas. Fieldwork teams found that the process was an invaluable step towards initiating relationships with PWID and developing the requisite skills for engaging with PWID. Team leaders found the experience useful in building their skills to implement HIV prevention interventions for PWID in challenging environments. During implementation, the study managers were frequently required to provide emotional and professional support to fieldworkers, some of whom currently or previously used drugs. The involvement of PWID (as fieldworkers and in CAGs and other consultations) increased levels of trust and access to a broader network of PWID, highlighting the importance of participatory planning and implementation of studies or services.

### Limitations

The assessment was not designed to assess risk practices or uptake of health services. Neither did the assessment differentiate between locations where PWID injected drugs or where they engaged in other activities. Challenges in measuring PWID mobility and reliance on “wisdom of the crowd” and “consensus” methods limit the robustness of our PWID size estimates. The potential extrapolation of the estimates to other areas with similar characteristics is limited, as we used non-random sampling techniques. Furthermore, contextual factors (e.g. drug availability, concentration of people, other factors contributing to movement and law enforcement activity) [50] influence drug use, and as such would influence injecting drug use location(s) in other wards and/or cities.

## Conclusions

Despite being restricted to a small number of wards in three cities, this study confirms that PWID are accessible, facing huge challenges to HIV prevention and are in need of increased attention, financing and support.

Structured formative assessments are useful to systematically map locations where PWID congregate, assess the availability of HIV prevention and health services, assess mobility and document experiences of law enforcement engagement. General community members (secondary key informants) who do not inject drugs do not have a good understanding of where injecting drug use takes place or where PWID are accessible. Formative assessments deepen the understanding of the injecting drug use context and provide an opportunity to establish trusting relationships with the PWID community and other stakeholders. The activities also build skills that service providers need to be able to effectively engage with and provide services to PWID—an essential preparatory step for service delivery.

This study provides the first local level size estimates of PWID in these cities. The range of estimates derived from the various methods used was wide but the use of many methods and inclusion of PWID community members may have increased the reliability of these estimates. In contrast to the service delivery data, other methods resulted in lower numbers of PWID for the selected wards. This could be linked to movement of PWID to locations where sterile injecting equipment was being provided (including PWID who congregate in public spaces outside of the mapped wards or who do not congregate with other PWID in public spaces), people transitioning to injecting, or other factors that need to be identified.

### Recommendations

Future formative assessments that make use of programmatic mapping techniques should consider focusing more on obtaining information from people who use drugs from the onset. Fieldwork teams should include people who currently or who have used or injected drugs to increase the efficiency of the formative assessment process. The provision of sterile injecting equipment along with brief harm reduction messaging should be considered for future formative assessments to increase the value of participation for PWID. Researchers and project planners embarking on formative assessments should consider classification of PWID locations, including locations where injecting occurs, and additional efforts to recruit females who inject drugs.

HIV prevention commodities, specifically sterile injecting equipment, and mechanisms for the safe disposal of used injecting equipment should be provided in the locations where PWID congregate or in locations where PWID can be accessed in Cape Town, Tshwane and eThekwini. Additional information on locations where females who inject drugs can be accessed to provide HIV prevention and harm reduction services in these cities is still needed. Considering the mobile nature of the PWID community, HIV prevention and harm reduction programmes should be flexible and delivered by mobile services and through peers to adapt to multiple service delivery routes in accordance with the mobile nature of PWID. Human rights violations should be documented as part of HIV prevention and harm reduction programmes to understand the impact on uptake of HIV prevention and harm reduction services by PWID and the concomitant health consequences thereof. Ward-based size estimates generated from formative assessments should be used as baseline estimates and for target setting for HIV prevention and harm reduction programming.

## Abbreviations

CAG: Community advisory group
GPS: Global positioning system
HIV: Human immunodeficiency virus
LI: Location identification
LV: Location verification
OST: Opioid substitution therapy
PEPFAR: United States’ President’s Emergency Plan for AIDS Relief
PWID: People who inject drugs
UNAIDS: Joint United Nations Program on HIV and AIDS
UNODC: United Nations Office on Drugs and Crime
WHO: World Health Organization
ZAR: South African Rand

## Footnotes

1 Exchange rate on 8 April 2017.

2 Validation was done at 10% of non-injecting locations in wards with less than five PWID locations and 5% of non-injecting locations in wards with five or more PWID

3 Service delivery commenced in June 2015, before data cleaning and analysis of the formative assessment was complete. As such, service delivery data from each city became available for comparison against the other estimates.
